# The relationship between effective personality and occupational health of lecturers: An empirical assessment in vietnam

**DOI:** 10.1016/j.heliyon.2023.e13231

**Published:** 2023-01-25

**Authors:** Nguyen Hai Thanh, Nguyen Ngoc Anh

**Affiliations:** Ho Chi Minh National Academy of Politics, Viet Nam

**Keywords:** Effective personality, Lecturers, Occupational health, Political schools, Satisfaction

## Abstract

There are gaps in research on the relationship between effective personality and occupational health of lecturers that need to be addressed. This study, therefore, explores the relationship between effective personality and occupational health of lecturers of provincial/city political schools in Vietnam. The survey is carried out in a convenient sampling method with 365 lecturers of provincial/city political schools in Vietnam. The questionnaires are provided to the participants of professional training courses at the Ho Chi Minh National Academy of Politics. The lecturers are willing to fill in the questionnaires at the same time. Descriptive, correlative, and linear regressive analyses are applied. The findings show that all elements of the effective personality structure have positive significant statistical impacts on enhancing self-efficacy, satisfaction, and cognitive affections while negatively causing exhaustion, musculoskeletal disorders, and voice disturbance of the lecturers' occupational health. Recommendations for promoting the effective personality of lecturers are provided. However, the study results are limited to the survey participants, who are lecturers of provincial political schools. Future research could expand the survey participants as well as elements of the effective personality structure and occupational health.

## 1 Introduction

Creating a healthy working environment has become an important topic for not only research organizations and scientists but also for the World Health Organization, which has brought this into its agenda [1]. There are four key dimensions of a healthy, safe and happy workplace, e.g. healthy working environment, effective psycho-social atmosphere, personal power, and effective personality of the staff at the workplace. This initial action disseminated knowledge and attracted attention to physical strength in the teacher’s workplace (Brouwers et al., 2011). However, just physical strength is not sufficient for a good working environment. Therefore, the research has expanded to other models on the working environment with a healthy psycho-social atmosphere, personal health power, and psychological dimensions [[2], [3], [4]].

This fact requires many researchers to develop and test theoretical frameworks on the impacts of psychological health on occupational health as well as their practical application [5]. There are analyses and assessments on causes of health degradation, increase in risks, and sickness due to psychological stress [6,7]. There are also chronological diseases and paints caused by psychological stress [8]. This explained that healthy mental status helps improve personal capability as well as occupational health and stable, healthy, and happy work [9,10]. When people are in declining occupational health, not only there is negative biological evidence such as respiratory and cardiovascular disorders but also negative psychological status triggerred [11]. Ruini [12] proved that there are positive relations between effective personality and human beings’ positive functions in occupational activities, which is critical for nurturing and maintaining occupational positiveness and satisfaction. Pedersen [13] expanded the research and explored international laws on calling for and mobilizing attention of interested organizations on creating, training, and maintaining effective personalities at work as well as searching for work satisfaction.

Creating and maintaining a healthy working environment and cooperative attitude at work is an important condition for enhancing productivity and ensuring the safety and happiness of each staff member. The importance is not what the person does but how he does it. Positive works show effective personalities [14]. Several studies stressed the increasing role of human resources in the school environment with a focus on mental and physical health strength. There are studies on psychological indicators such as trust, respect for explaining the teachers’ achievements [15,16], the need to work, ability to control the work balance regarding occupational health, exhaustion or risks at work for teachers [17]. Many research even explore elements of the working environment. However, personality elements are missing. Therefore, there are calls for strengthening personal resources to improve occupational health, work organization manners, personal capacity, and power development. This help regulates and controls psychological variables to enhance the teachers’ quality [18].

In class lecturing, personal resources have been identified and acknowledged to be factors of effective personality structure [19] and occupational health in lecturing activities [20]. Effective psychology and occupational interest are important factors for lecturers to gain abundant energy in the lecturing profession and enjoy becoming lecturers. These are evidence of lecturers with effective personalities [21]. The lecturers’ positive emotional status helps reduce the risks of unnecessary stress [22]. Furthermore, effective psychological stages are optimal for intellectual and professional development, which help lecturers nurture positive professional relationships [23,24].

Other studies explained the impacts of psychological factors on the physical and mental health status, of which the most obvious result is emotional exhaustion such as depression with negative psychological signs appear and quickly reduce teaching productivity [25]. The lecturers’ declining occupational health is reflected by slow body reactions and less convincing voices. These proportionately increase with the rising psychological impacts [26]. In contrast, when the lecturers have healthy and happy mental status, their professional capability and performance, as well as their job satisfaction increase [27]. In addition, these studies explore the impacts on the quality of professional communication. The result shows that effective personality increases the quality of communication with colleagues and occupational health while negative personality signals reduce occupational health [[28], [29], [30]].

While there are many research on the relationship between effective personality and occupational health in general and lecturers’ health in certain contexts and countries in particular, no such study is carried out in Vietnam so far. There are 63 provincial political schools throughout Vietnam with a large number of lecturers. The regulations on the development of standard political schools set out criteria for the development of quality lecturers [31]. Therefore, the main focus of this study will be analysis of the relationship between effective personality and occupational health of lecturers at provincial political schools. This will enhance the effective personality of lecturers, and thereby improve their occupational health and satisfaction as well as prevent and reduce negative impacts.

Effective personality plays an important role in not only the psychological life but also in the protection of lecturers’ health. Lecturers in political schools do not seem to pay adequate attention to knowledge and the development of effective personalities. In the context of the Vietnamese education reform in general and the development of standard political schools in particular, the focus is still very much on the content, teaching syllabus, and lecturers’ professional qualifications [32]. To meet these standards and evaluation criteria, lecturers face significant pressure in lecturing their classes. This has a large impact on lecturers’ psychology and health. However, effective personality, which is an important element in the protection of lecturers’ health, has not been either mentioned or paid adequate attention to. Even though there is evidence of the impacts of effective personality on lecturers’ occupational health, this issue in political schools seems to be a gap. Therefore, research and evaluation of the impacts of effective personality on lecturers’ occupational health in political schools in Vietnam are critically needed.

There are political schools in 63 provincies/cities of Vietnam with large number of lecturers. All of lecturers in these 63 political schools are focused on political training and research. This is different with other universities, where many lectures and research on various sciences are conducted. In addition [31], issued regulation No. 11-QĐ/TW on standard political schools, which requires the lecturers to meet professional and personal standards and reputation. Furthermore, provincial political schools play important role in enhancing leadership quality of local authorities, consolidating their operation. This is the rationale for this study to analyse and explore the relationship between positive personality and occupational health of lecturers at political schools.

## 2 Literature review

### 2.1 Effective personality

Effective personality is an integrated model of personal capability and social experience, which forms a quadrilateral structure: self-esteem, laboral self-realization, resolute self-efficacy, and social self-realization. These angles connect and reflect the positive extent of personality [19,33]. Among these angles, self-esteem reflects the perception of personal values, which include self-evaluation of knowledge, emotion, social intelligence, and other people’s assessment of advantages and limitations. Labor self-realization emphasizes the link between knowledge and personal achievements through daily efforts. Resolving self-efficacy means one’s capacity to respond effectively to the challenges in their life, including decision-making and implementation to achieve the set objectives. Social self-realization is the communication skills, determination, and empathy with others in social relations. These angles are also taken as a model of effective personality, which guarantees good personal physical and mental status [34,35]. [36] argue that effective personality is reflected by positive emotion, which contributes largely to the worker’s maturity and growth in the workplace. Therefore, the workplace should be stimulating for the workers’ creativity and innovation. Workers with effective personalities tend to face challenges more actively and look at situations more calmly and flexibly thanks to their good learning ability. As a result, they will solve the problems more effectively [[37], [38], [39]].

### 2.2 Occupational health

At the 12th meeting in 1995, the International Labor Organization, World Health Organization, and Committee on Occupational Health announced a joint declaration that stated: “Objective of occupational health is to promote and maintain the highest level of physical, mental health and social welfare in all sectors; prevent the workers’ health decline caused by harmful factors at the workplace. Based on these elements, to maintain a professional working environment that is relevant to workers’ physical and mental conditions. This means creating adaptability of the work for human beings and each person at the workplace” [40]. However, discrepancies and gaps in understanding occupational health exist, particularly in developing countries [41]. The concept of occupational health aims to protect workers from occupational dangers and harms [42], but in reality, the occupational health principles omit the workers that are not on the list of protected occupations [43]. Therefore, the goals of occupational health should be to protect workers in all occupations against negative effects of occupational dangers and harms on the workplace. There are certain levels of occupational risks and impacts in all sectors and occupations, depending on the occupational nature and working environment. The recommendations for improvement in occupational health regulations stress strict regulations on the workers’ working conditions and working space to ensure occupational health safety of workers of different nationalities [43].

### 2.3 Job satisfaction

Job satisfaction is defined as the extent of love and positive emotion through workers’ objective evaluation of all or some work-related aspects [44,45]. Job satisfaction is viewed as popular evidence of workers’ productivity, achievements, and rewards [46]. Moreover, job satisfaction also comes from the workers’ objective evaluation and the conditions that stimulate workers’ positive attitude at work [47]. The more staff evaluate the job satisfaction, the higher leadership effects. Vice versa, the fewer staff evaluate job satisfaction implies declining productivity and working conditions [48]. Christen, Iyer, and Soberman [49] argue that job satisfaction is reflected in a quadrilateral model including job-related factors, problems with role perception, job performance, and firm performance. Job satisfaction is even seen as the emotional status [[50], [51], [52]]. Most of the studies tested the impacts of workers’ job satisfaction on their motivation, productivity, and subsequently the operational effectiveness as well as better performance and sustainability of organizations.

### 2.4 Relationship between effective personality and occupational health

Effective personality has a decisive role in the development of good mental health, which is an important condition for maintaining good physical and mental health and happy life. These, in turn, are the positive power sources for workers’ motivation, innovation, and optimal productivity. The evidence of the relationship between effective personality and occupational health, therefore, has been confirmed. Effective personality, together with other factors, helps induce optimism, prevent occupational risks, and increase workers’ occupational health and happiness [53]. There have been recommendations for enhancing occupational health as well as effective personality, even paying attention to each factor of effective personality, to enhance the workers’ immunity to negative occupational effects and improve performance [18,54,55] explore in-depth each aspect of effective personality, e.g. self-esteem, labor self-realization, resolute self-efficacy, and social self-realization. They confirm the following impacts on the occupational health of lecturers: exhaustion, satisfaction, voice disturbance, musculoskeletal disorders, cognitive affectations, and self-efficacy. On the other hand, the impacts of effective personality have been tested positive on occupational health as follows: self-efficacy and satisfaction and effective personality will reduce or eliminate negative signs of occupational health such as exhaustion, cognitive affections, musculoskeletal disorders, and voice disturbance. Hence, the research results are consistent with the positive impacts of effective personality on occupational health, and the reduction or elimination of negative signs of occupational health. This study aims to test the impacts of effective personality on the occupational health of lecturers at political schools in Vietnam by exploring each dimension of effective personality in relation with the occupational health of lecturers at political schools. This helps fill in the research gap in this area.

## 3 Research objective and hypothesis

### 3.1 Key research objectives include

Exploring signs of the effective personality of lecturers at provincial political schools in Vietnam.

Exploring signs of a relationship between effective personality and occupational health of lecturers at provincial political schools in Vietnam.

Checking on the relationship between effective personality and occupational health of lecturers at provincial political schools in Vietnam.

### 3.2 The following hypotheses will be tested

H1 Effective personality has an effective relationship with signs of improved occupational health of lecturers: self-efficacy, satisfaction, and cognitive affections.

H2 Effective personality has a negative relationship with signs of declining occupational health of lecturers: exhaustion, musculoskeletal disorders, and voice disturbance.

## 4 Research methodology

### 4.1 Study design

This study follows the horizontal descriptive design, correlative and deducive analysis, which is consistent with one-time research [56]. The questionnaire on effective personality [19] and the questionnaires on occupational health [20] were distributed to the interviewed lecturers. Collected data was run by correlative and regression tests. The participants were informed about the confidentiality and ethical standards of this study, which have been approved by the Ethics Committee of Ho Chi Minh National Academy of Politics. The lecturers volunteered and agreed to fill in the questionnaires.

### 4.2 Participants

Prior to the survey, all the lecturers have been well informed about the content of regulation No. 11-QD/TW on standard political schools issued by the Secretariat of the Communist Party of Vietnam [31]. The political schools had discussions on requirements for lecturers of standard political schools. Participating lecturers were consent and willing to respond to questionnaire, expecting that this study is an important science-based channel for policy-making bodies to issue relevant regulations and remmunerations for lecturers at political schools under rising pressure and workload to satisfy requirements of standard political schools. The samples were taken in clusters during their training period at the same time.

The lecturers of the provincial political schools were selected for survey. The total number of lecturers at provincial political schools is 1.992 [57], therefore the sample size is 333. The convenient sample selection approach was applied, based on accessibility to the participants. The survey time was from November 15, 2021 to April 27, 2022. The interviewed lecturers are currently working at provincial political schools, which are under the provincial party committees’ management. The provincial political schools are mandated to provide education and training courses for the leading and management cadres of the political system at the grassroots level as well as local government officials on political theories and administration, party and government policies, legal and professional knowledge, research and practical experience review and summary, etc. The final sample size was 365 lecturers, representing lecturers from all provincial political schools. The distribution of participants is presented in Table 1 below.

**Table 1:** Characteristics of study participants.

Variables	Category	Number of participants (365)	Percentage of participants
Gender	Male	193	52.88
	Female	172	47.12
Age	Under 30	81	22.19
	31–35 years	94	25.75
	36–40 years	106	29.04
	Above 40	84	23.01
Teaching seniority	1–5 years	14	3.84
	6–10 years	43	11.78
	11–15 years	80	21.92
	16–20 years	93	25.48
	21–25 years	67	18.36
	26–30 years	45	12.33
	Above 30 years	23	6.30
Education	Bachelor	44	12.05
	Master	273	74.79
	Doctor	48	13.15
Income	Purely from salary	257	70.41
	Salary and other non-salary and unstable sources	85	23.29
	Stable non-salary income sources	23	6.30
Political study degree	Without political study degree	30	7.40
	Junior political study degree	92	24.66
	Political bachelor degree	40	6.85
	Graduate political study degree	203	61.10

In Table 1, the distribution of the samples is consistent with demographic category and sample size of the variables gender, age, teaching seniority, education, income and political study degree, which suffifiently represent total 1992 lecturers.

### 4.3 Instruments

The Questionnaire on Effective Personality [19] and occupational health [20] were used in this study. Questionnaire of Effective Personality-Adults (CPE-A) includes 30 items, which are integrated into a quadrilateral structure: self-esteem, labor self-realization, resolute self-efficacy, and social self-realization. Self-esteem consists of 8 items and has Cronbach's alpha coefficient of 0.82. These items clarify the awareness of appearance, emotional ability, social knowledge, and acceptance of personal limitations. Labor self-realization consists of 8 items and has Cronbach's alpha coefficient of 0.79, focusing on the evaluation of knowledge, resource distribution for personal interests, efforts, motivation, and expectation of achievable results. Resolute self-efficacy consists of 5 items, has Cronbach's alpha coefficient of 0.85, and analyses the way the lecturers respond actively and effectively before teaching situations in their profession. Social self-realization consists of 9 items, have Cronbach's alpha coefficient of 0.87, and identifies communication skills, determination, sympathy, and the way a person establishes and maintains his/her relationships in professional activities. The Likert ranking system with 5 points divided from 1 to 5 with: 1 = never; 2 = few times; 3 = sometimes; 4 = many times; 5 = always, has been tested and confirmed the results [58].

Questionnaire of Lecturers Health (CSD) [20], which consists of 23 items, has 6 dimensions: (1) exhaustion, with 3 items, has Cronbach's alpha coefficient 0.86, identifies the lecturers’ physical and mental health after a certain period of doing research or teaching; (2) satisfaction, with 5 items, has Cronbach's alpha coefficient 0.78, identifies physical and mental status at work place; (3) voice disturbance, with 3 items, has Cronbach's alpha coefficient 0.83, assesses the body’s physical status after the teaching process through the voice; (4) musculoskeletal disorders, with 3 items, has Cronbach's alpha coefficient 0,81, assesses physical characteristics, mainly through backbone and back; (5) cognitive affectations, with 4 items, has Cronbach's alpha coefficient 0.84, assesses the concentration ability signs; (6) self-efficacy, with 5 items, has Cronbach's alpha coefficient 0.77, identifies the lecturers’ awareness ability on personal achievable results as well as personal professional capability. Responses of the lecturers are rated based on the Likert 5-point ranking from 1 to 5: 1 = strongly disagree; 2 = disagree; 3 = neither agree nor disagree; 4 = agree; 5 = strongly agree. This questionnaire is considered a good and relevant tool for assessing lecturers’ occupational health by monitoring current health status [18]. All results have a reliability coefficient of Cronbach's alpha >0.7, which demonstrates optimum outcomes in both questionnaires on effective personality and occupational health [59]. The design structure of the questionnaires and Cronbach's alpha coefficient are presented in Table 2.

**Table 2:** Questionnaire design, Cronbach's alpha.

Variable		Instrument authors	No. of items	Order of items	Cronbach's alpha	Measurement scale
Effective personality	Self-esteem	[19]	8	3, 7, 11, 15, 19, 23, 26, 29	0.82	1 = N, 5 = A 5-point scale
	Labor self-realization		8	1, 5, 9, 13, 17, 21, 24, 27	0.79	1 = N, 5 = A 5-point scale
	Resolute self-efficacy		5	4, 8, 12, 16, 20	0.85	1 = N, 5 = A 5-point scale
	Social self-realization		9	2, 6, 10, 14, 18, 22, 25, 28, 30	0.87	1 = N, 5 = A 5-point scale
Lecturers’ Occupational Health	Exhaustion	[20]	3	7, 12, 17	0.86	1 = SD, 5 = SA 5-point scale
	Satisfaction		5	6, 10, 13, 18, 23	0.78	1 = SD, 5 = SA 5-point scale
	Voice disturbance		3	8, 14, 16	0.83	1 = SD, 5 = SA 5-point scale
	Musculoskeletal disorders		3	2, 5, 21	0.81	1 = SD, 5 = SA 5-point scale
	Cognitive affectations		4	3, 9, 11, 20	0.84	1 = SD, 5 = SA 5-point scale
	Self-efficacy		5	1, 4, 15, 19, 22	0.77	1 = SD, 5 = SA 5-point scale

Note: SD = Strongly disagree, SA = Strongly agree; N = Never; A = Always.

The first two questionaires were translated from orginal Spanish languague into Vietnamese with relevant adaptation to Vietnamese culture. As such, the questions satisfy the study’s purpose and the lecturers’ awareness and needs. As participating lecturers have been involed in discussions on effective personality and occupational health, the questionnaire on effective personality and occupational health is relevant with political schools in Vietnam. The questions are also translated into English and enclosed as commented by the reviewer.

The questionnaire was distributed on-site and the participants filled them in immediately. We translated the original questionnaire from Spanish into Vietnamese and made relevant adjustments to the context of political schools. The Cronbach’s alpha test results in Table 2 show that these are scientifically satisfactory.

### 4.4 Data analysis

The data collected from questionaires on effective personality and the questionnaire on occupational health was analyzed by the SPSS 26.0 system, which was developed by Cano et al. [19], and Fernández-Puig [20] respectively. The descriptive statistics method applied in both questionnaires to explore the lecturers’ occupational health extent. In addition, the descriptive statistics method applied to identify Cronbach's alpha coefficient, Pearson correlation, and linear multiple regression analysis.

In this study, effective personality is the independent variable and the lecturers’ occupational health is the dependent one. In addition, bivariate Pearson correlations were carried out to assess the relationship between the two models, between the overall and specific aspects of effective personality and occupational health. Linear regression was run to identify the proportion that effective personality accounts for in the occupational health of lecturers at provincial political schools. On the other hand, Bonferroni adjustment was also conducted to assess the impacts of effective personality on the occupational health of lecturers.

The model fit test in CFA is used, in which the CMIN/df index measures the absolute fit of the model, with Chi-square/df (CMIN/df), CMIN/df ≤ 3 is good, CMIN/df ≤ 5 is acceptable [60]. Comparative fit index (CFI) is used in the model to measure the fit of statistical results, CFI ≥0.9 is good, CFI ≥0.95 is very good, and CFI ≥0.8 is acceptable (CFI ranges from 0 to 1) is considered one of the best statistic indexes [61,62], in which 0.90 is the minimum value to protect the model [63]. In addition, the goodness of fit index (GFI) is measured from 0 to 1. The GFI ≥0.9 is good, and GFI ≥0.95 is very good [61,64]. Standardized root-mean-square (SRMR) measures the number of errors of the model, the good fit has values below 0.05 [65] and the normed fit index (NFI) measures the statistical decline in statistics χ^2^ of the model compared to the basic model, with minimum NFI value of 0.90 [18].

## 5 Results

The statistical analysis and quantitative description through lecturers’ responses in the questionnaires on effective personality and occupational health show that the assessment of effective personality and occupational health has a higher mean than the theoretical mean in all cases. Moreover, standard skewness and kurtosis are between ±1.96 values is significant at 5%, which means that the tested results are accepted [66].

Table 3 shows a lower theoretical mean than the empirical mean in both the questionnaire on effective personality and occupational health of lecturers. In addition, the values of self-efficacy, satisfaction, and cognitive affections are greater than that of exhaustion, voice alterations, and musculoskeletal affections in occupational health (Table 3).

**Table 3:** Description of effective personality and occupational health of lecturers.

Variables: CPE-A and CDS		No of items	Theoretical mean	Empirical mean	Standard deviation	Minimum	Maximum	Skewness	Kurtosis
CPE-A	Self-esteem	8	25.38	30.16	4.18	17	38	0.32	0.63
	Labor self-realization	8	24.54	33.75	3.56	20	31	−0.26	0.17
	Resolute self-efficacy	5	16.17	20.04	2.84	9	23	0.37	0.48
	Social self-realization	9	28.35	34.94	4.93	18	42	−0.41	0.29
	Total	30	94.44	118.89	11.85	64	130	0.28	0.27
CDS	Exhaustion	3	9.63	9.94	3.25	5	13	0.41	0.58
	Satisfaction	5	16.23	21.05	3.57	6	23	−0.46	0.39
	Voice alterations	3	9.74	9.95	3.16	4	14	−0.23	0.67
	Musculoskeletal affections	3	9.46	10.26	3.07	5	13	0.29	−0.56
	Cognitive affections	4	13.07	14.18	3.19	6	19	0.37	0.23
	Self-efficacy	5	15.58	21.16	3.18	9	23	−0.53	0.71
	Total	23	73.71	86.54	11.96	35	105	0.16	0.35

Results found in relationships between the signs of effective personality and the signs of occupational health of lecturers, the Pearson correlative analysis is conducted when the variables are within the acceptable range, to test the diviation and kurtosis. In addition, the Pearson correlative analysis shows positive and significant correlation between signs of effective personality and signs of occupational health of lecturers (p < 0.05 and p < 0.01).

Table 4 shows that 6 CPE-A factors have significant relationship with CDS factors, in which: satisfaction correlates with social self-realization, self-esteem, labor self-realization and resolute self-efficacy with results r = 0.72**, 0.74**, 0.73** and 0.69** respectively, overall r = 0.71** (p < 0.01). Positive correlation between cognitive affections of occupational health with social self-realization, self-esteem, labor self-realization and resolute self-efficacy of effective personality with r of 0.61**, 0.73**, 0.65**, 0.57** respectively and overall r = 0.64** (p < 0.01). The self-efficacy positively correlates with social self-realization, self-esteem, labor self-realization and resolute self-efficacy with coefficient of 0.75**, 0.70**, 0.71**, 0.71** (p < 0.01) respectively and overall r = 0.67** (p < 0.01). On the contrary, exhaustion has negative relationship with social self-realization, self-esteem, labor self-realization and resolute self-efficacy of effective personality, with r coefficient of −0.53**, −0.61** (p < 0.01), 0.47* (p < 0.05), −0.54** (p < 0.01) respectively and overall r = −0.62** (p < 0.01). The voice alterations effectively correlates with social self-realization, self-esteem, labor self-realization and resolute self-efficacy of effective personality, with r coefficient of 0.63**, 0.57**, 0.54** (p < 0.01), 0.42* (p < 0.05) respectively and overall r = 0.39* (p < 0.05). Finally, musculoskeletal affections in occupational health correlate positively with social self-realization, self-esteem, labor self-realization, and resolute self-efficacy of effective personality, with r coefficient of 0.36*, 0.45*, 0.38*, 0.46 × respectively and overall r = 0.41* (p < 0.05). In general, the occupational health of lecturers has a positive correlation with each factor of effective personality as well as the relationship between the occupational health of lecturers and effective personality is significant statistically, with r coefficient of 0.63**, 0.67**, 0.52**, 0.48** and 0.54** (p < 0.01) respectively (Table 4).

**Table 4:** Correlations between effective personality lecturer’s health.

Variables: CPE-A and CDS		CPE-A				
		Social self-realization	Self-esteem	Labor self-realization	Resolute self-efficacy	Total
CDS	Exhaustion	−0.53**	−0.61**	−0.47*	−0.54**	−0.62**
	Satisfaction	0.72**	0.74**	0.73**	0.69**	0.71**
	Voice alterations	−0.63**	−0.57**	−0.54**	−0.42*	−0.39*
	Musculoskeletal affections	−0.36*	−0.45*	−0.38*	−0.46*	−0.41*
	Cognitive affections	0.61**	0.73**	0.65**	0.57**	0.64**
	Self-efficacy	0.75**	0.70**	0.71**	0.71**	0.67**
	Total	0.63**	0.67**	0.52**	0.48**	0.54**

Note: *p < 0.05 (one tailed); **p < 0.01 (two tailed).

In addition to the test on the correlation between effective personality and occupational health of lecturers as well as each factor of effective personality structure and each factor of occupational health structure, linear regression was carried out to assess the impacts of each factor of effective personality structure on each factor of occupational health structure of the lecturers.

Regression in Table 5 was carried out based on self-efficacy. The assumption for conducting multiple linear regression satisfies the test, with a positive significant correlation (p < 0.01), which supports the linear relationship between self-efficacy and the variables of effective personality. In addition, the Durbin–Watson statistics are used to check whether there is autocorrelation in residuals. Durbin-Watson value = 0.33, which is within the range of 0 < d < 1, shows positive correlation, satisfying independence requirements, meaning that H1 is accepted. The Variance inflation factor (VIF) values, which measure correlation and the strength of correlation between variables, are within the range from 1.16 to 4.58 and less than 10, which means there is no multilinearity while the remaining is standardized according to covariate distribution. 10.13039/100014337Furthermore, the test result Kolmogorov-Smirnov fails to support the standard assumption (p < 0.01). On the other hand, regression shows that self-efficacy correlates with self-esteem (β = 0.62**, R^2^ = 0.56), labor self-realization (β = 0.53**, R^2^ = 0.55), resolute self-efficacy (β = 0.58**, R^2^ = 0.55) and social self-realization (β = 0.53**, R^2^ = 0.55), which are important forecast elements explaining the variance of 55.1%. Therefore, the regression results show that self-efficacy in occupational health explains changes in self-esteem, labor self-realization, resolute self-efficacy and social self-realization (Table 5).

**Table 5:** Regression of the influence of effective personality factors on self-efficacy.

Dependent variable	Independent variable	Unstandardized coefficients (B)	Standardized coefficients *(β)*	95% Confidence Interval		R square *(R)*	Adjusted R square *(R*^2^*)*	*R*^2^ Corrected	Change at *R*^2^
				Lower	Upper				
Self-efficacy	(Constant)	6.73		2.37	5.26	0.63	0.55	0.55	0.00**
	Self-esteem	0.62	0.56**	0.14	0.74				
	Labor self-realization	0.57	0.53**	0.32	0.67				
	Resolute self-efficacy	0.58	0.55**	0.27	0.48				
	Social self-realization	0.55	0.53**	0.21	0.45				

**Bonferroni correction (p-value 0.01/3 tests = 0.002).

In Table 6, the regression in satisfaction in occupational health of lecturers. In general, except for normality, all assumptions for multiple linear are satisfied. The regression results show the statistical significance of variables (p < 0.05), which reflects a comprehensive linear relationship. The Durbin–Watson statistics were used to check whether there is autocorrelation in residuals. The Durbin-Watson = 0.747, which is within the range of 0 < d < 1, shows positive correlation, satisfying the independence, meaning H1 is accepted. VIF values, which measure correlation and the strength of correlation between variables, are within the range from 0.01 to 4.53, which are less than 10, meaning that there is no multicollinearity [67], while the remainder is standardized according to covariate distribution. Furthermore, the test result Kolmogorov-Smirnov fails to support the standard assumption (p < 0.01). As such, the regression results show the impacts of the satisfaction factor on self-esteem, labor self-realization, resolute self-efficacy, and social self-realization, which are important forecast factors. The independent variables explain 51.6% of the variance (p < 0.01), which supports an effective relationship between these factors (Table 6).

**Table 6:** Regression on impacts of factors in effective personality on factor satisfaction.

Dependent variable	Independent variable	Unstandardized coefficients (B)	Standardized coefficients *(β)*	95% Confidence Interval		R square *(R)*	Adjusted R square *(R*^2^*)*	*R*^2^ Corrected	Change at *R*^2^
				Lower	Upper				
Satisfaction	Constant	2.81		1.64	4.03	0.53	0.51	0.51	0.00**
	Self-esteem	0.62	0.57**	0.19	0.41				
	Labor self-realization	0.55	0.53**	0.14	0.23				
	Resolute self-efficacy	0.58	0.51**	0.21	0.36				
	Social self-realization	0.57	0.52**	0.27	0.39				

**Bonferroni correction (p-value 0.01/3 tests = 0.002).

Regression in Table 7 was carried out on factor cognitive affections. The results in Table 9 support the overall positive correlations and statistically significant between factor cognitive affections and factors of self-esteem, resolute self-efficacy, social self-realization, and labor self-realization in occupational health of the lecturers (*p* < 0.05). This is a comprehensive linear relationship. The Durbin–Watson statistics were used to check whether there is autocorrelation in residuals. The Durbin-Watson = 0.94, which is within the range of 0 < d < 1, shows positive correlation, reflecting well the independence characteristics, meaning H1 is accepted. In addition, the VIF values, which measure correlation and the strength of correlation between variables, lie within the range from 0.57 to 4.53, which confirms the variance is less than 10, therefore no multicollinearity. Furthermore, the Kolmogorov-Smirnov test result fails to support the standard assumption (p < 0.01). Hence, regression results show positive correlation between signs of factor cognitive affections and self-esteem (β = 0.54**, R^2^ = 0.53), labor self-realization (β = 0.36*, R^2^ = 0.53), resolute self-efficacy (β = 0.51**, R^2^ = 0.53), and social self-realization (β = 0.51**, R^2^ = 0.53), which are important forecast factors. The independent variables explain well the variance of 53.5% of dependent variables while the remaining 46.5% are explained by variables outside the model and random errors, which all show a relatively strong positive relationship (p < 0.01), exept for factor labor self-realization that has fewer impacts but also correlates positively (p < 0.05) (Table 7).

**Table 7:** Regression on impacts of factors in effective personality on cognitive affections.

Dependent variable	Independent variable	Unstandardized coefficients (B)	Standardized coefficients *(β)*	95% Confidence Interval		R square *(R)*	Adjusted R square *(R*^2^*)*	*R*^2^ Corrected	Change at *R*^2^
				Lower	Upper				
Cognitive affections	(Constant)	18.25		13.52	19.64	0.58	0.53	0.53	0.00**
	Self-esteem	0.56	0.54**	0.31	0.47				
	Labor self-realization	0.38	0.36*	0.16	0.35				
	Resolute self-efficacy	0.59	0.51**	0.14	0.32				
	Social self-realization	0.60	0.57**	0.09	0.21				

**Bonferroni correction (p-value 0.01/3 tests = 0.002); *Bonferroni correction (p-value 0.05/3 tests = 0.018).

**Table 8:** Regression of the influence of factors in effective personality on the factor on exhaustion.

Dependent variable	Independent variable	Unstandardized coefficients (B)	Standardized coefficients *(β)*	95% Confidence Interval		R square *(R)*	Adjusted R square *(R*^2^*)*	*R*^2^ Corrected	Change at *R*^2^
				Lower	Upper				
Exhaustion	Constant	9.21		5.57	10.43	0.58	0.51	0.51	0.00**
	Self-esteem	−0.63	−0.55**	−0.33	−0.48				
	Labor self-realization	−0.52	−0.50**	−0.24	−0.51				
	Resolute self-efficacy	−0.57	−0.52**	−0.19	−0.45				
	Social self-realization	−0.52	0.51**	−0.26	−0.45				

**Bonferroni correction (p-value 0.01/3 tests = 0.002).

**Table 9:** Regression of the influence of factors in effective personality structure on musculoskeletal disorders.

Dependent variable	Independent variable	Unstandardized coefficients (B)	Standardized coefficients *(β)*	95% Confidence Interval		R square *(R)*	Adjusted R square *(R*^2^*)*	*R*^2^ Corrected	Change at *R*^2^
				Lower	Upper				
Musculoskeletal disorders	(Constant)	16.73		9.78	17.52	0.11	0.43	0.43	0.00**
	Self-esteem	−0.53	−0.51**	−0.17	−0.46				
	Labor self-realization	−0.62	−0.57**	−0.23	−0.42				
	Resolute self-efficacy	−0.56	−0.52**	−0.21	−0.61				
	Social self-realization	−0.54	−0.53**	−0.32	−0.53				

**Bonferroni correction (p-value 0.01/3 tests = 0.002).

Regression in Table 8 was carried out on the factor exhaustion. Overall, the assumptions for multiple linear regression are supported. The regression results show a significant correlation between factor exhaustion in occupational health of the lecturers and factors of self-esteem, labor self-realization, resolute self-efficacy with 95% of confidence, the significance of 5% (p < 0.01), which show a comprehensive linear relationship. The Durbin–Watson statistics are used to check whether there is autocorrelation in residuals. The Durbin-Watson = 3.97, which is within the range from 3 < d < 4, meeting the requirements on independence. Yet, it shows negative correlation, meaning H2 is accepted. In addition, VIF values lie within 0.03–4,12, which are less than 10, showing that there is no multicollinearity. The Kolmogorov-Smirnov test result fails to support the standard assumption (p < 0.01). Hence, regression results show statistically significant and negative relationship between factor exhaustion with self-esteem (*β* = −0.55**, *R*^2^ = 0.51), labor self-realization (*β* = −0.52**, *R*^2^ = 0.51), nhân tố resolute self-efficacy (*β* = −0.57**, *R*^2^ = 0.51) and social self-realization (*β* = −0.51**, *R*^2^ = 0.51), which are important forecast factors that explain variance of 51.6% (Table 8).

Regression in Table 9 was carried out on factor musculoskeletal disorders in the occupational health of the lecturers. The results in Table 8 show a negative correlation between factor musculoskeletal disorders with factors of self-esteem, labor self-realization, resolute self-efficacy, and social self-realization in the effective personality of the lecturers, no factor is omitted (p < 0.01), which implies a comprehensive linear relationship. In addition, the Durbin-Watson value = 3.58, which is within the range of 3 < d < 4, satisfying the requirements for independence characteristics. Yet it shows negative correlation, meaning H2 is accepted. VIF values are from 0.78 to 4.35, which are all below 10, meaning that there is no multicollinearity. The Kolmogorov-Smirnov test result fails to support the standard assumption (p < 0.01). Hence, the regression results show a negative correlation between factor musculoskeletal disorders in occupational health of lecturers with factors self-esteem (β = −0.51**, R^2^ = 0.43), labor self-realization (β = 0.57**, R^2^ = 0.32), resolute self-efficacy (β = 0.52*, R^2^ = 0.32) and social self-realization (β = −0.53**, R^2^ = 0.43) in effective personality. These are important forecast factors and explain the variance of 43.2% (Table 9).

Finally, regression in Table 10 was carried out on the factor voice disturbance. There is an average negative correlation between the factor of voice disturbance in effective personality structure with the factors of self-esteem, labor self-realization, resolute self-efficacy, and social self-realization in the occupational health of the lecturers (p < 0.05). All assumptions for linear regression, except normality, are satisfied. There are comprehensive linear relationships. Durbin-Watson = 3.59, which is within the range of 3 < d < 4, hence satisfying the requirements for independent characteristics. Yet, it shows negative correlation, meaning H2 is accepted. VIF values are from 0.13 to 3.56, which are less than 10, meaning that there is no multicollinearity. The Kolmogorov-Smirnov test result fails to support the standard assumption (p < 0.05). In summary, regression results show a negative relationship between the factor voice disturbance in occupational health and self-esteem (*β* = −0.31*, *R*^2^ = 0.32), labor self-realization (*β* = −0.43*, *R*^2^ = 0.32), resolute self-efficacy (*β* = −0.48*, *R*^2^ = 0.32), and social self-realization (*β* = −0.45*, *R*^2^ = 0.32), which all are important forecast factors and explain for variance of 32.7% (Table 10).

**Table 10:** Regression of the influence of effective personality factors on voice disturbance.

Dependent variable	Independent variable	Unstandardized coefficients (B)	Standardized coefficients *(β)*	95% Confidence Interval		R square *(R)*	Adjusted R square *(R*^2^*)*	*R*^2^ Corrected	Change at *R*^2^
				Lower	Upper				
Voice disturbance	(Constant)	12.56		5.39	14.74	0.45	0.32	0.32	0.02*
	Self-esteem	−0.34	−0.31*	−0.36	−0.48				
	Labor self-realization	−0.47	−0.43*	−0.08	−0.35				
	Resolute self-efficacy	−0.53	−0.48*	−0.26	−0.52				
	Social self-realization	−0.50	−0.45*	−0.17	−0.36				

*Bonferroni correction (p-value 0.05/3 tests = 0.018).

The results of the questionnaires further strengthen and confirm the possible relationships between the signs of effective personality and the occupational health of the lecturers. Both questionnaires on effective personality and occupational health show consistent results with the model, including the CMIN/DF = 2.56, 2.37 ≤ 3, which is good [60], both GFI = 0.90, 0.92; CFI = 0.91, 0.89; TLI = 0.87, 0.95; NFI = 0.91, 0.93 in Table 10 show confirmatory factor analysis between 0 and 1, which are good (Hairet al., 2009) (Table 11). In addition, SRMR = 0.07, 0.05, which is < 0.08 [61] and RMSEA = 0.29, 0.31 in both models <0.05 [66].

**Table 11:** Testing the model’s relevance.

	Model 1		Model 2	
CMIN/DF	2.56		2.37	
GFI	0.90		0.92	
CFI	0.91		0.89	
TLI	0.87		0.95	
NFI	0.91		0.93	
SRMR	0.07		0.05	
RMSEA	0.02		0.04	
	**Effective Personality**	**Occupational Health**	**Effective Personality**	**Occupational Health**
Composite reliability	0.87	0.81	0.86	0.84
Average variance extracted	0.61	0.53	0.67	0.72
Maximum shared variance	0.54	0.49	0.63	0.62
MaxR(H)	0.83	0.88	0.91	0.92
Effective Personality	0.73	0.78**	0.75	0.79**
Occupational Health	0.62**	0.57	0.63**	0.76

Note: **p < 0.01.

Table 11 also shows composite reliability (CR) in both questionnaires on effective personality and questionnaire on occupational health in model 1 và model 2 with CR > 0.7 and average variance extracted (AVE) in both model 1 and model 2 > 0.5, which guarantees the convergence. In addition, maximum shared variance (MSV) is < AVE, MaxR(H) > 0.8 showing the differentiation is in place [68]. Furthermore, both model 1 and model 2 confirm a positive correlation between effective personality and occupational health of the lecturers (p < 0.01) (Table 11).

## 6 Discussion

This study analyses and discusses on relationship between effective personality and occupational health of lecturers at provincial political schools as well as each dimension of effective personality structure and occupational health. In this relationship, occupational health is an independent variable while the factors of effective personality structure are dedepent variables. Two hypotheses have been tested and confirmed. These are: effective personality has positive impacts on self-efficacy, satisfaction, and cognitive affections, meaning that the H1 is supported. Studies of Healey and Grossman [69], Rychlak and Slife [70] show positive correlation between effective personality với cognitive affections. On the other hand, effective personality has negative impacts on exhaustion, musculoskeletal disorders, and voice disturbance in occupational health of lecturers [18,71]. As such, H2 is accepted.

The results from two survey questionnaires on effective personality and occupational health provide the basis for confirming that effective personality is an important factor that has a significant impact on enhancement of the occupational health of lecturers through the following signs: self-efficacy, satisfaction, and cognitive affections. This is consistent with the previous finding that effective personality helps protect the occupational health of the lecturers through self-efficacy, and satisfaction [18]. However, this study also shows that effective personality also contributes to the protection and improvement of factor cognitive affections [71]. In addition, the regression analysis confirms that stronger signs of effective personality have greater positive impacts on factors of self-efficacy, satisfaction, and cognitive affections. Hence, hypothesis H1 has been accepted.

Moreover, an effective personality has not only positive impacts on the occupational health of the lecturers but also on human development, happiness, and prosperity [72]. The findings in this study further confirm the results of earlier research, which prove this in the teaching environment of high schools [18]. This is also applied to lecturers at political schools and expanded to factor cognitive affections. When the lecturers are confident with their professional capability, satisfied with their current job, trusted and appreciated by colleagues, dedicated to making efforts and striving for better performance, and socially skilled, their occupational health will be improved through factors of self-efficacy, satisfaction, and cognitive affection. In addition, these will also create opportunities for their professional development and enable happiness and prosperity. The linear regression analysis supports this positive relationship between effective personality and occupational health of the lecturers.

In addition to the positive impacts of effective personality on occupational health, the findings in this study also confirm its negative impacts on factors exhaustion, musculoskeletal disorders, and voice disturbance. The regression analysis confirms the strong negative correlation between effective personality and health declining factors including exhaustion, musculoskeletal disorders, and voice disturbance. The results are statistically significant. The H2 therefore has been accepted. This further strengthens the argument that teachers that have a stronger effective personality (meaning they are satisfied with their current job, positively emotional with the existing working environment, happy with relationships with colleagues, content with the leadership, guidance), the more likely that they are less affected by exhaustion, musculoskeletal disorders, and voice disturbance. On the contrary, declining effective personality might be one of the causes that lead to negative signs of the factors exhaustion, musculoskeletal disorders, and voice disturbance in the occupational health of the lecturers.

These results are consistent with Ruini [12], and Kobasa [73], which indicate that individuals that have higher effective personality grading have better health, stronger will and determination, better and more active response to challenges, better control, and more willingness to change themselves, and less negative impacts from outside. Moreover, a high negative emotional extent will cause negative consequences within the personality structure and possibly will decrease social functions [74]. Therefore, effective personality has positive impacts not only on the occupational health of the lecturers but also on the protection of the occupational health of the lecturers at political schools.

There are many research findings and discoveries on general impacts of personality recently [75], as well as the impacts of effective personality on teachers’ occupational health [76,77]. Effective personality has not only been considered an important factor that has positive impacts on health but also an integral factor, which stimulates teachers in their professional life. The findings in this study therefore are consistent and further strengthen the previous findings of earlier research. Effective personality has an important role in promoting proactive spirit, job dedication, and productivity of the lecturers at political schools. In return, better occupational health helps maintain and strengthen an effective personality. There are always bilateral positive relationships between effective personality and occupational health.

Furthermore, there are common aspects of the findings on occupational health of the lecturers in showing important motivations to promote activities related to occupational health [78], positive activities, in turn, stimulate positive emotion, which is related to working motivation of the lecturers [79]. The productive, highly motivated lecturers always have positive emotions, psychology, and personality. The lecturers’ positive emotions and attitudes are decisive factors for their working attitude, and job satisfaction [16]. Observations on positive relationships between effective personality and occupational health are found in Reilly et al. [15], Caprara et al. [16], which confirm that the higher the teachers have signs of effective personality, the higher working motivation, spirit, and enthusiasm they have.

On the other hand, the research confirms a negative relationship, even relatively strong, between signs of declining occupational health of lecturers and low job satisfaction with effective personality. In this study, the lecturers that have the strongest signs of effective personality have negative collaboration with three factors in occupational health, e.g. exhaustion, musculoskeletal disorders, and voice disturbance. The lower these signs are, the higher job satisfaction there will be. This result has been found by Brouweret et al. [17], showing that the stronger signs of effective personality are reflected, the better negative signs of occupation health will be decreased. In addition, the high level of factors such as exhaustion, musculoskeletal disorders, and voice disturbance demonstrate that the lecturers have lower personal power including self-esteem, labor self-realization, resolute self-efficacy, and social self-realization, as well as a lower level of occupational health. Moreover, the high level of exhaustion, musculoskeletal disorders, and voice disturbance are likely to cause lecturers to lose their confidence, decrease productivity and lower their expectations for success, therefore greater negative psychology. This has been pointed out by Ferradás et al. [80], that when lecturers have low self-esteem, they show ineffective communication capacity, awareness, attention, and observation as well as an easy distraction [81]. It is hard to find exhaustion in productive, successful, happy lecturers [80].

## 7 Limitations

First, this study is designed to gain awareness and reflection from lecturers at provincial political schools. Therefore, the results and conclusions might not be comprehensive and applicable to all lecturers at universities and teachers at high schools. Future research can expand the research target group through quantitative analysis in more diversified groups. Second, this study uses only two questionnaires, namely the Questionnaire of Effective Personality-Adults (CPE-A) and Questionnaire of Lecturers Health (CSD) therefore may not cover sufficiently all aspects of effective personality and occupational health of the lecturers. As a result, other signs, and factors of personality and occupational health may be out of the scope of this study. As such, it is necessary to have additional research with other questionnaires to have more comprehensive and more satisfactory answers about the relationship between effective personality and occupational health as well as job satisfaction in lecturing activities.

## 8 Future research potential

This study is designed horizontally, therefore the research in the future may be designed vertically to go more in-depth into the development of analyzed variables. As a result, this can establish a cause-and-effect relationship.

Other research in the future may expand to other groups that are not lecturers at political schools but are lecturers at universities, school teachers, and other target groups in various cultural contexts of the country with larger sample sizes to collect more sufficient data for summarizing empirical activities.

This study may use human personality development programs by expanding factors of personality structure as well as occupational health to research different dimensions of positive personality and enhance the productivity and teaching capacity of not only lecturers but also other groups in various contexts and cultures.

To generate better evidence for firm consolidation of results of this study, it is possible to expand interview methods, and portrait research methods and subsequently help the lecturers nurture and maintain effective personalities to have good psychology, healthy emotion, and strong physical health.

## 9 Conclusion

The results of this study confirm the effective relationship between effective personality and the signs of occupational health including self-efficacy, satisfaction, and cognitive affections of the lecturers at political schools. In addition, these positive impacts help reduce negative signs in the occupational health of the lecturers including exhaustion, musculoskeletal disorders, and voice disturbances. These findings reveal the impacts of effective personality on the occupational health of lecturers in teaching activities. Therefore, effective personality can be seen as an important factor to enhance productivity, improve teaching capacity and develop personal health resources in teaching occupation, in schools as well as in the training of teachers. Paying attention to effective personality and teachers’ mental health will make their professional activities become more effective and healthy.

The findings of this study reflect the Vietnamese context, with occupation charateristics of lecturers at political schools. It therefore migh not be relevant for other universities in Vietnam or in other countries. The findings reflect the relationship between effective personality and occupational health of lecturers that teach theoretical political study in Vietnam.

## Declarations

### Author contribution statement

Nguyen Hai Thanh: Conceived and designed the experiments; Performed the experiments; Contributed, reagents, materials, analysis tools or data; Analyzed and interpreted the data; Wrote the paper.

Nguyen Ngoc Anh: Conceived and designed the experiments; Contributed reagents, analysis tools or data; Analyzed and interpreted the data.

### Funding statement

This research did not receive any specific grant from funding agencies in the public, commercial, or not-for-profit sectors.

### Data availability statement

Data included in article/supplementary material/referenced in article.

### Declaration of interest’s statement

The authors declare no competing interests.

## Lecturers Health Questionnaire (CSD)

1. I have the ability to be creative and agile in my teaching activity
2. My back suffers from the activity I do
3. At times, I lack the concentration to carry out tasks
4. I feel capable of making decisions
5. I suffer from lumbago
6. I enjoy my daily tasks
7. After the working day I find myself without strength
8. I feel hoarse or dysphonic
9. Sometimes I have the impression that I get obsessed and think about issues that I could resolve at another time without any problem
10. I have a good time at work
11. There are times when I have more distractions than usual
12. I feel physically exhausted at the end of my workday
13. I feel satisfied with my way of doing things
14. My voice tires easily
15. When I wake up, I feel like going to work
16. My neck feels itchy after work
17. At work, I get very exhausted
18. If I could choose again, I would be a teacher again
19. I am satisfied with my contribution to the school
20. Lately I have a lack of memory
21. I often feel pain in the area of the neck
22. When I finish a job, I am often happy with the results
23. I am very happy with my work

### Effective Adult Personality Questionnaire

CPE-A: Adults.

1. My successes in my profession are due to my effort and dedication
2. I have few friends because it is difficult for me to deal with others
3. I feel very good about my physical appearance
4. When I have to make a decision, I carefully plan what I am going to do.
5. My success in a task is due to the fact that I strive to do a good job
6. I make friends easily
7. There are many things about myself that I would like to change if I could.
8. When I have a problem I try to see the positive side of it
9. I have a good ability to work
10. My failures in relationships with others are due to my inability to make friends.
11. In general I feel satisfied with myself
12. To make a decision, I gather all the information I can find.
13. I consider myself a good worker
14. I think that knowing myself as I am, I will have problems in my relationships with others
15. I accept myself as I am, with my qualities, limitations, and defects
16. I control my emotions well
17. I work because I like to overcome the challenges that tasks present me
18. I believe, with certainty, that I will be successful in my relationships with others
19. I have many qualities to be proud and satisfied with myself
20. When I experience a failure I try to learn from that experience
21. My success at work is due to my personal ability
22. My successes in relationships with others are due to my ability to make friends
23. I manage to make almost everything work for me
24. When I have a problem I dedicate time and effort to solve it
25. My successes in relationships with others are due to the fact that they are the ones who take the initiative
26. I think I am a valuable person to others
27. I am convinced that I will be successful when I work
28. I think that my failure in relationships with others is due to the fact that most people do not like me.
29. I think I have the talent to make things work
30. I feel comfortable sharing with other people
